# Stable Expression of Antibiotic-Resistant Gene *ble* from *Streptoalloteichus hindustanus* in the Mitochondria of *Chlamydomonas reinhardtii*

**DOI:** 10.1371/journal.pone.0035542

**Published:** 2012-04-17

**Authors:** Zhangli Hu, Zhun Fan, Zhonglin Zhao, Jun Chen, Jiancheng Li

**Affiliations:** 1 Shenzhen Key Laboratory of Marine Bioresource and Eco-environmental Science, College of Life Science, Shenzhen University, Shenzhen, People's Republic of China; 2 College of Sciences, Henan Agricultural University, Zhengzhou, People's Republic of China; Ben-Gurion University of the Negev, Israel

## Abstract

The mitochondrial expression of exogenous antibiotic resistance genes has not been demonstrated successfully to date, which has limited the development of antibiotic resistance genes as selectable markers for mitochondrial site-directed transformation in *Chlamydomonas reinhardtii*. In this work, the plasmid pBSLPNCB was constructed by inserting the gene *ble* of *Streptoalloteichus hindustanus* (*Sh ble*), encoding a small (14-kilodalton) protective protein into the site between TERMINVREP-Left repeats and the *cob* gene in a fragment of mitochondrial DNA (mtDNA) of *C. reinhardtii*. The fusion DNA-construct, which contained TERMINVREP-Left, *Sh ble*, *cob*, and partial *nd4* sequence, were introduced into the mitochondria of the respiratory deficient *dum-1* mutant CC-2654 of *C. reinhardtii* by biolistic particle delivery system. A large number of transformants were obtained after eight weeks in the dark. Subsequent subculture of the transformants on the selection TAP media containing 3 ìg/mL Zeomycin for 12 months resulted in genetically modified transgenic algae MT-Bs. Sequencing and Southern analyses on the mitochondrial genome of the different MT-B lines revealed that *Sh ble* gene had been integrated into the mitochondrial genome of *C. reinhardtii*. Both Western blot, using the anti-BLE monoclonal antibody, and Zeomycin tolerance analysis confirmed the presence of BLE protein in the transgenic algal cells. It indicates that the *Sh ble* gene can be stably expressed in the mitochondria of *C. reinhardtii*.

## Introduction

Unlike the genetic transformation of the nucleic and chloroplast genomes, genetic modification of the mitochondrial genome is still very limited in higher plants and animals [1], [2], with successful stable expression of foreign functional genes in the mitochondria only demonstrated in *Saccharomyces cerevisiae* [3], [4]. Besides the *Saccharomyces cerevisiae*, *Chlamydomonas reinhardtii* is a good choice for mitochondrial studies, as it can undergo mitochondrial transformation with homologous genes [1], [5], [6] or heterologous protein [7]. It has distinct advantages in comparison to higher plants as it is unicellular, haploid, is amenable to tetrad analysis, and its' three genomes are theoretically subject to specific transformation [8]. However, the stable expression of exogenous antibiotic resistance genes in *C. reinhardtii* mitochondria have not been routinely performed to date, and an effective selectable marker for screening the successful mitochondrial transformation has not yet been found.

To construct a transformation and screening system, we have developed a recombinant plasmid containing *Sh ble* gene, which was isolated from *Streptoalloteichus hindustanus* and confers Zeomycin resistance. Zeomycin is a copper-chelated glycopeptide antibiotic, structurally related to the group of bleomycin and phleomycin type antibiotics, and it is toxic to both prokaryotic and eukaryotic cells. The antibiotic is effective on most aerobic cells and is therefore useful for selection of cells that express the *Sh ble* gene in bacteria, eukaryotic microorganisms, plants and animal cells. As there is no cross resistance with other currently used animal cells markers, this antibiotic can also be used to isolate clones resistant to other selecting agents. In some cases, the *Sh ble* protein can be fused to other proteins (such as the green fluorescent protein) for visual screening and drug selection of transfected eukaryotic cells [9]. The *Sh ble* gene displays interesting characteristics which make it a useful marker for the nuclear transformation in *C*. *reinhardtii* [10], [11], [12].

In this study, a *Sh ble* gene was integrated into the mitochondrial genome of *C*. *reinhardtii* cc-2654 by homologous recombination. Subsequently, Zeomycin resistant protein was expressed in the mitochondria of *C*. *reinhardtii*. To our knowledge, this is the first report of stable expression of an exogenous antibiotic-resistant gene in the mitochondria of photosynthetic organism. It provides the opportunity to develop the *Sh ble* gene as a selectable marker for mitochondrial site-directed transformation in *C*. *reinhardtii*.

## Results

### Construction of mitochondrial expression vector containing *Sh ble* gene

**Figure 1:**
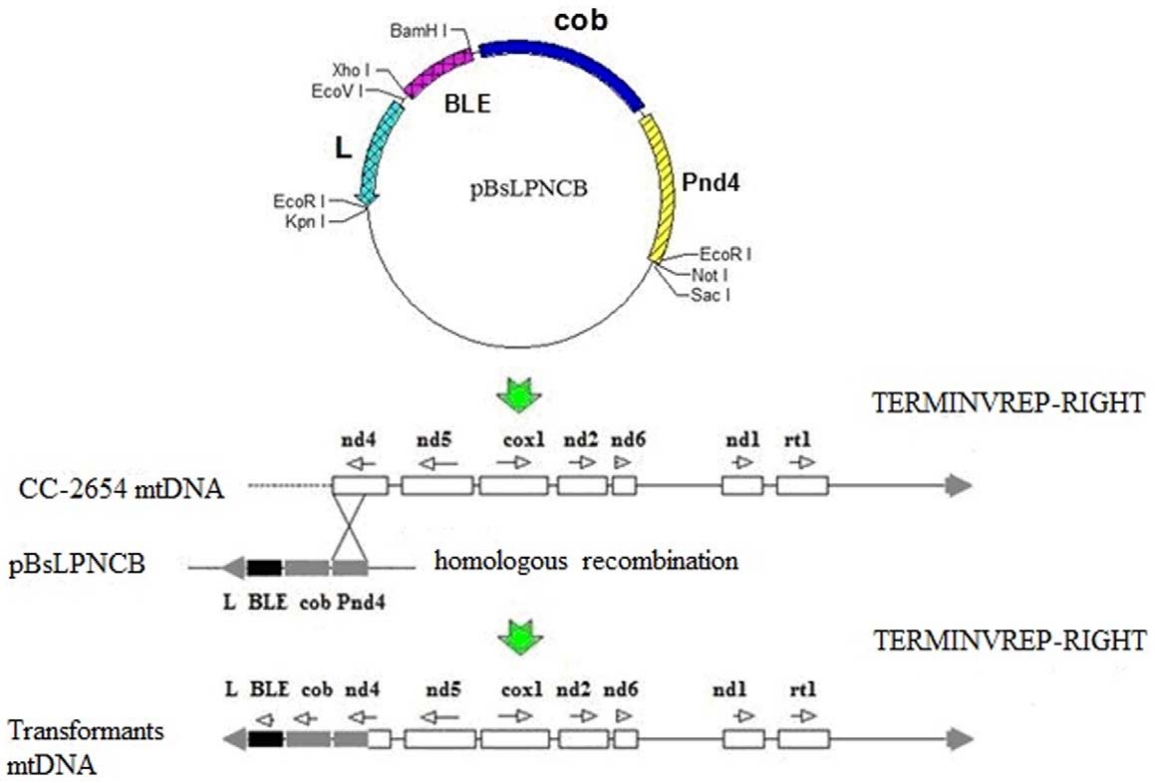
Schematic diagram of homologous recombination events between pBsLPNCB and CC-2654 mtDNA. The dotted line represents the deletion part of mtDNA in CC-2654 relative to wild-type CC-124. The empty boxes are the mitochondrial genes and the empty arrows above the genes indicate their transcription directions. The crossed solid lines denote the homologous recombination region between the CC-2654 mtDNA and expression vector pBsLPNCB.

The expression vector containing a resistance marker was constructed by inserting the *Sh ble* gene into pBsLPNC. The left arm and PNC sequences were amplified from the mitochondrial genome of *C*. *reinhardtii* CC-124 and inserted into pBluescript II SK to create pBsLPNC. The *Sh ble* gene was amplified and cloned into plasmid pBsLPNC. In the resulting plasmid pBsLPNCB, the *Sh ble* gene was flanked by the left arm and PNC fragments. The DNA fragment PNC contains the *cob* gene and a part of the *nd4* gene. The fragment containing the *Sh ble* gene was integrated to the mitochondrial genome of *C. reinhardtii* cc-2654 by homologous recombination mediated by this part of *nd4* gene (Figure 1).

### Mitochondrial transformation and screening of transgenic algae MT-Bs

**Figure 2:**
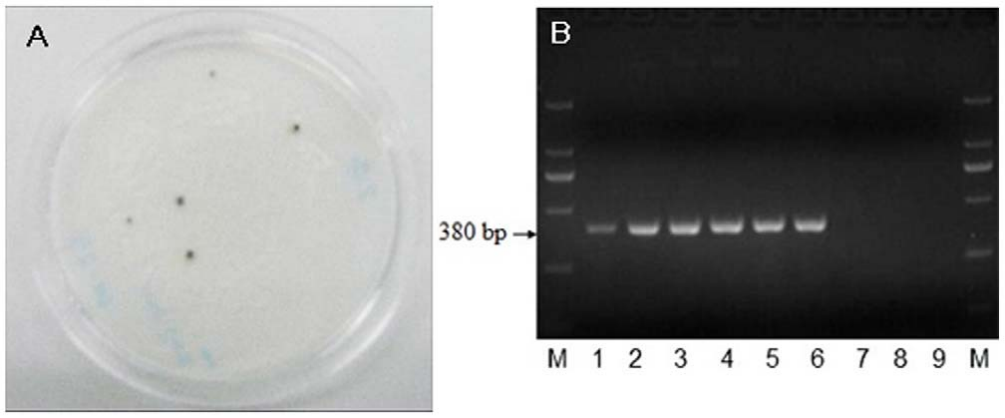
Growth of transformants MT-Bs on the TAP media with 3 µg/mL Zeomycin (A) and PCR analysis with B1/B2 primers (B). M, DL2000 marker; lanes1-6, different clones of MT-B; lanes 7–8, negative controls CC-2654 and CC-124; lane 9, water used as negative control.

The respiratory deficient *dum-1 mt^−^* CC-2654 mutant of *C. reinhardtii* was used as a recipient strain. The CC-2654 mutant can not grow in dark, because the left fragment containing TERMINVREP-Left and *cob* gene of its mtDNA was truncated. The *Sh ble* gene was sandwiched between the TERMINVREP-Left arm sequence and *cob* gene from the mitochondrial genome of wild-type strain *C. reinhardtii* CC-124. The fusion DNA-construct was introduced into the mtDNA of *C. reinhardtii* CC-2654 by biolistic particle delivery system. The transformants were obtained in the dark after 8 weeks. Subsequent subculture of the transfromants on the selection TAP media containing 3 ìg/mL Zeocin for 12 months resulted in genetically modified transgenic algae named *C. reinhardtii* MT-B (Figure 2A). Actually, one week of subculturing on selective medium was enough to obtain homozygous transformants. 12 months was used to reveal whether long period would influence transformants. We found that the 12 months' transformants and one week' transformants had no obvious genetic differentiation. The presence of transgenes in transformants was further investigated. For comparison, DNA samples isolated from wild-type CC-124, respiratory-deficient mutant CC-2654 and transgenic MT-B were used for PCR amplification of *Sh ble* (about 380 bp) using primers B1/B2. The expected PCR bands were found in the MT-B transformants only (Figure 2B).

### Analysis of the *ble* integrated site in the mitochondrial genome of MT-B

To locate the *ble* gene, a 4 kb fragment of mtDNA was analyzed by DNA sequencing. Results showed that left arm sequence was from 1–504 bp, *Sh ble* was 557–931 bp and the PNC fragment containing *cob* and *nd4* was 931–2737 bp. The sequence from 2738–3491 bp was the other part of nd4 gene in the mtDNA of cc-2654. This demonstrated that the construct fragment was integrated to the mitochondrial genome of *C. reinhardtii* cc-2654 by homologous recombination mediated by part of the *nd4* gene.

**Figure 3:**
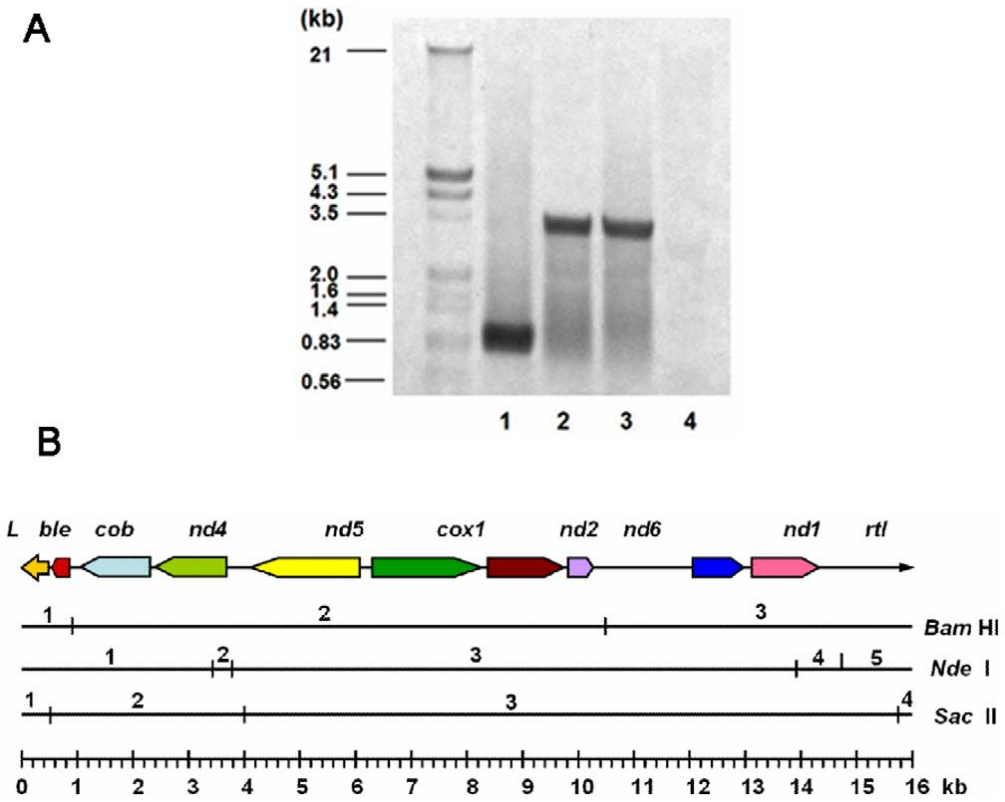
Analysis of the *Sh ble* gene integrated site in the mitochondrial genome of MT-B. A: The Southern hybridization of *C. reinhardtii* MT-B with the ble-probe; B: Restriction map of the mitochondrial genome of *C. reinhardtii* MT-B showing the positions of all mapped genes and restriction sites. Line 1,2, 3: Total DNA of MT-B were digested with *Bam* HI, *Nde* I and *Sac* II, respectively, Line 4: the genome of CC-2654 digested with *Nde*I was used as control.

Total DNA was digested with *Bam* HI, *Nde* I and *Sac* II, respectively, and blotted with the *ble*-probe. A single band of approximately 0.8 kb was present in *Bam* HI digested DNA. *Nde* I and *Sac* II digests also showed a single band of approximately 3.3 kb (Figure 3). The result matched the restriction map of mitochondrial genome of MT-B. Only one southern hybridized band with three restriction enzymes demonstrated that there was just one copy of *Sh ble* in the transformant MT-B. It indicated that *Sh ble* gene was integrated to the mitochondrial genome of *C. reinhardtii* cc-2654, with no nuclear genome insertion.

### *Sh ble* gene transcripts in transformant MT-B

**Figure 4:**
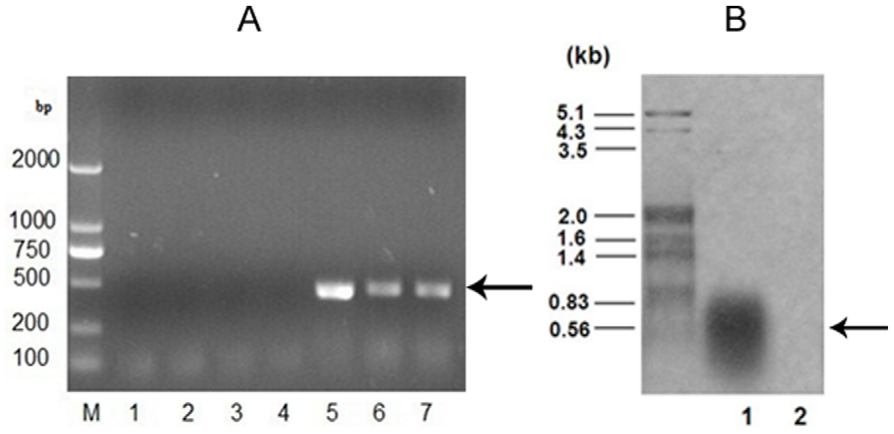
*Sh ble* gene transcripts in transformant MT-B. Panal A is the RT-PCR analysis of MT-B strains and PCR amplification with total RNA. M: DL2000 marker, Lane 1: cDNA of negative control CC-2654 used as template; 2–4: total RNA isolated from different MT-B strains used as template; lane 5–7: cDNA of different MT-B strains used as template. Panal B is the Northern blot analysis of *C. reinhardtii* MT-B. Lane 1, total RNA of MT-B was detected with *Sh ble* probe; lane 2. total RNA of CC-2654 was used as negative control.

Total RNA of MT-B was isolated and reverse-transcripted to cDNA. Results of the RT-PCR showed a single band of 380 bp corresponding to the molecular weight of *Sh ble* (Figure 4A, lane 5–7).

Northern blot analysis was used to detect the steady-state level of the transcript of *Sh ble* (Figure 4B). Total RNA was probed with a probe specific for the *Sh ble* transcript. A significant signal band of 0.38 kb was detected, corresponding to the predicted size of the *ble* transcript. This proved a good indicator for the transcriptional activity of *Sh ble* in transgenic MT-B.

### Expression of the BLE protein in the mitochondria of *C. reinhardtii*

**Figure 5:**
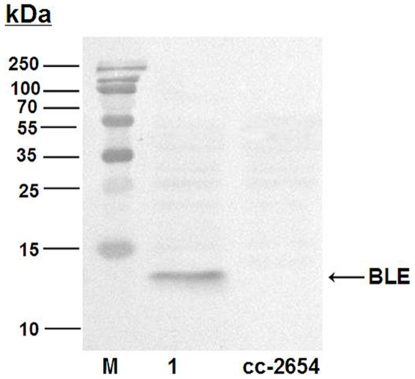
Western blotting analysis of the transformants MT-B using monoclonal anti-BLE antibody. The soluble protein of CC-2654 (2) were used as control, the soluble protein of transformants MT-B (1) was detected by Western blotting.

Western blot analysis was performed to detect the *Sh ble* gene product at a protein level using monoclonal anti-BLE antibody. Total soluble protein of the transgenic algae was used for the analysis and a protein band with 13.7 kDa was detected in transgenic algae as expected (Figure 5). This indicated that the transgene *Sh ble* was stably expressed in mitochondria of the transformant MT-B.

### Resistance and Sensitivity of transgenic algae MT-Bs to Zeomycin in *C. reinhardtii*

**Figure 6:**
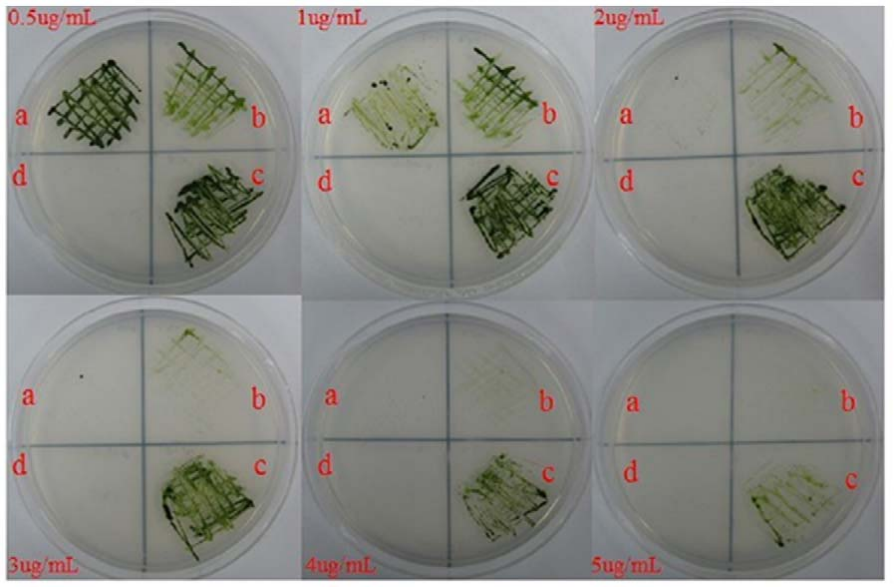
Sensitivity analysis of transgenic strains MT-Bs to Zeomycin. a: wild-type strain CC-124; b: respiratory deficient strain CC-2654; c: transgenic strain MT-B; d: empty control.

Sensitivity of C. *reinhardtii* transformants to Zeomycin was observed using different concentrations of Zeomycin added to the solid TAP plate. 2 µg/ml Zeomycin was found to completely inhibit the growth of *C. reinhardtii* CC-124 and CC-2654. However, the transgenic strain MT-B contained *ble* gene grew well on TAP agar plate containing 3 µg/ml Zeomycin. 5 µg/ml Zeomycin inhibited visible the growth of *C. reinhardtii* MT-B after incubation (Figure 6). The data suggested that mitochondrial expression of the *Sh ble* gene rendered the transformants certain levels of resistance against Zeomycin.

### The expression levels of the exogenous *Sh ble* and the mitochondrial genes in transgenic MT-B

**Figure 7:**
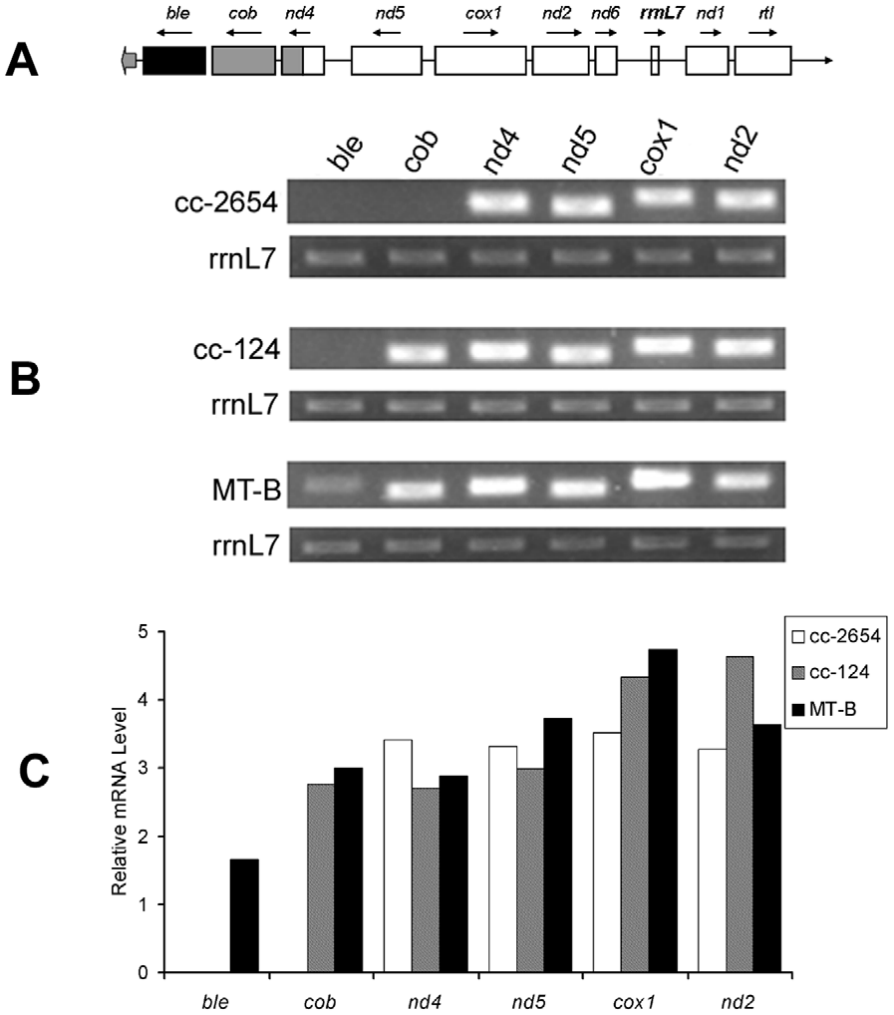
The expression levels of the *Sh ble* gene and the mitochondrial genes in transgenic MT-B. A: The gene organization of MT-B mitochondrial genome: B: RT-PCR results of *Sh ble* gene and the mitochondrial genes with rrnL7 as internal control; C: Relative mRNA levels analysis using BIO-RAD image software.

The semi-quantitative RT-PCR methods were used to investigate the expression differences of *Sh ble*, *cob*, *nd4*, *nd5*, *cox1* and *nd2* among transgenic MT-B, cc-2654 and wild type cc-124. The results showed: (1) the insertion of *Sh ble* gene did not significantly affect other mitochondrial genes expression. (2) Theoretically, the *Sh ble* and other mitochondrial genes should belong to constitutive co-expression, which was initiated by the bi-directional promoter between *nd5* and *cox1* [13]. However, our results showed that the expression level of exogenous *Sh ble* was less than other mitochondrial genes in transgenic MT-B (Figure 7). The possible factors that effected the expression of *Sh ble* gene included the codon bias, promoter and intron [14], [15].

## Discussion

*C*. *reinhardtii* is the only photosynthetic organism whose mitochondria can be genetically modified with homologous mtDNA [13]. Early attempts to develop a mitochondrial transformation system for the green alga *C.reinhardtii* relied on the complementation of respiratory deficient mutant cc-2654 with the *cob* gene encoding apocytochrome b from wild type cc-124. After integration with the transgene *cob*, genetically modified respiratory deficient mutants can grow in the dark and the transformants can consequently be easily screened. The transformant selection could not be applied to higher multicellular organisms, because these organisms can not survive due to respiratory defect. It was reported that two plasmids were co-transformed by biolistic bombardment, one plasmid carried the nuclear selectable marker, and the other contained a mitochondrial transgene. At first, transformants were screened by the selectable marker of nuclear transformation under light illumination, then they were transferred into dark condition for further screening of mitochondrial transformants, but this approach did not work well [16]. So far, no effective antibiotic resistance genes are stably expressed in mitochondria of photosynthetic organisms, which limits the development of mitochondrial genetic transformation.

Zeomycin is an antibiotic that is sensitive in yeast, green algae, plant and animal cells. The *ble* gene from *Streptoalloteichus hindustanus* can be stably expressed in the nuclear genomes of mammalian cells [17], plants [18], yeast [19] and green alga *C*. *reinhardtii* [20], so that transformants of these organisms become Zeomycin resistant and can be screened using Zeomycin-containing selection media. However, although stable expression of foreign genes has been achieved [21], and many marker genes for transformant screening have been found in the nuclear or chloroplast genomes of *C. reinhardtii* [22], [23], the mitochondrial expression of exogenous marker genes has not been successfully expressed to date. In this study, a fusion DNA-construct, which contained TERMINVREP-Left, *Sh ble*, *cob*, and partial *nd4* sequence, was introduced into the mitochondria of respiratory deficient *dum-1* mutant CC-2654 of *C. reinhardtii*. Proof that the DNA-construct had been inserted into the mtDNA of respiratory deficient mutant cc-2654 was demonstrated as the transformant MT-B was able to grow on the TAP selection media containing 3 ìg/mL Zeomycin under dark conditions. The sequence analysis of left 4 kb fragment of the MT-B mtDNA furtherly confirmed that the *Sh* ble gene was integrated into the mtDNA by homologous recombination. Previous work has found mitochondrial pseudogenes in the nucleus of eukaryotes, with many copies of nuclear counterparts of mtDNA were found in nuclear DNA [24]. In this study the southern blot detection of RFLPs with three restriction enzymes clearly demonstrated that there was only one copy of *Sh ble* in the transformant MT-B (Figure 3). It indicated that *Sh ble* gene was only integrated to the mitochondrial genome of transformant MT-B and none of the *Sh ble* gene had been inserted into the nuclear mtDNA pseudogenes of *C. reinhardtii* by homologous recombination.

The transgenic algae MT-B kept the Zeomycin resistance after it was cultured in TAP media for two years (Figure 6). We never observed a loss of Zeomycin resistance in transgenic algae maintained on non-selective condition. It showed that the expression of the *Sh ble* gene was stable in the mitochondria of transgenic algae. To our knowledge, this is the first report of stable expression of an exogenous antibiotic resistance gene in the mitochondria of *C.reinhardtii*.

It is unclear how algal cells acquire the Zeomycin resistance by the expression of BLE protein inside the mitochondria of transformants MT-B. Zeomycin is a DNA intercalating agent that can destroy all three cellular genomes of algal cells. Theoretically then, the expression of the BLE protein in the mitochondria would protect only the mitochondrial DNA, with no protection provided to the nuclear and the chloroplastic DNA. However, our experimental results showed that the transgenic MT-Bs could grow on TAP agar plate containing 3–5 µg/ml Zeomycin (Figure 6). This indicates that the BLE protein is exported from the mitochondria in the transgenic MT-B. The putative explanations are that: within the mitochondria exists a specific protein export mechanism [25], [26], BLE is a small molecule of the heterologous protein (13 kD), it can be delivered outside mitochondria or out of the algae by the protein export mechanism to create Zeomycin resistance. Further detailed pathway studies will be required.

A cell of *C. reinhardtii* contains 30–50 copies of the mitochondrial genome [16]. In a transformation event, not every mitochondrial genome is integrated with the foreign gene fragment, and heterogeneous cells containing both transformed and non transformed mitochondria exist. As a transformant selection process proceeds, separations of heterogeneous mitochondrial genome occured and homogenous transformant cells were finally obtained. In the *C. reinhardtii* chloroplasts, this homogenization process of transformant cells is quite fast [27], but the process of mitochondrial homogenization is very slow in the respiratory deficient mutant of *C. reinhardtii*; heterogeneous transformant clones still remained after incubation in a dark environment for 2–3 months [1]. However, TAP plates containing Zeomycin were used to enhance the pressure of transformant selection and homogenization in this study. When the transgenic clones were subcultured on selection media containing 3 µg/ml Zeomycin for one week, our results showed that all transformant lines were homoplasmic (Figure 2). This suggests that Zeomycin resistance selection was able to accelerate the homogenization process of mitochondrial transformants.

The resistance to Zeomycin was much weaker in the transgenic strains MT-B than that previously reported in algae [11], [12], mammalian cells [17], plants [18], yeast [19] or bacteria [28]. *Sh ble* gene expression allowed the growth of transgenic bacteria at 25–50 µg/mL Zeomycin on a low-salt growth medium. Transgenic yeast, plants and mammalian cells can grow at 100 µg/mL of Zeomycin. The transgenic MT-B that contained *Sh ble* gene in mitochondrial genome could only grow on TAP agar plate containing 3–5 µg/ml Zeomycin. However, the lower mitochondrial expression of *Sh ble* gene was enough to be used to generate stable mitochondrial transformants by selection against Zeomycin. The expression of the *Sh ble* gene based on the mitochondria could be developed as a resistance marker of expression system for transgenic research and expression of other kinds of recombinant proteins in mitochondria of *C. reinhardtii*.

Our results showed that the transgenic alga MT-B could produce BLE proteins after subculturing for 24 months. It indicates that the gene *Sh ble* can be stably expressed in the mitochondria of *C. reinhardtii*. As the full sequence of *Sh ble* contains only 375 bp, it is suitable and feasible to be used as a selectable marker for the mitochondrial genetic transformation of *C. reinhardtii*. In particular it makes it possible to integrate a transgene into different sites within the *C. reinhardtii* mitochrodrial genome by site-specific recombination, not just limited to the left site [6]. Our results shed light on mitochondrial reverse genetics studies and genetic engineering in the photosynthetic organisms.

## Materials and Methods

### Strains and Growth Conditions

*C. reinhardtii* respiratory deficient strains *dum-1 mt^−^* CC-2654 and wild-type strain CC-124 were obtained from *Chlamydomonas* Genetic Center (c/o Dr. Elizabeth H. Harris, Department of Botany,Duke University,Durham, NC27706, USA). All strains were grown on TAP medium containing 1% agar at 22°C under the following lighting conditions; [a 12∶12 hour light (80–100 µmol m^−2^ S^−1^): dark].

### PCR amplification and sequencing analyses

The PCR amplifications were performed according to standard protocols [29]. Based on the sequence information of the *C. reinhardtii* mitochondrial genome (GenBank accession number U03843) and *S. hindustanus* Ble gene sequence (GenBank accession number A31898.1), the oligonucleotide primers for the PCR amplification were the following: L1 (5′-AACTGCAGCCTCGAGGGATATCTATTTTGCATTGACACA C-3′, *Pst* I, *Xho*I and *Eco*RV recognition sites underlined), L2 (5′-GGGGTACC- CGGAATTCACTACGCATGCCTAAG-3′, *Kpn*I and *Eco*RI recognition sites underlined), PNC1 (5′-TTGCGGCCGCCGGAATTCTGTACTATTGAAACTAGG- AGGCA-3′, *Not*I and *Eco*RI recognition sites underlined) and COB 2 (5′-AA CTGCAGCCCCGGGCGCGGATCCTTAGTTGGTTTGAGTACCGTGG-3′, *Pst* I, *Sma*I and *Bam*HI recognition sites underlined), B1 (5′- CGCGGATCCGCGATG- GCCAAGTTGACCAGT-3′, *BamH* I recognition sites underlined), B2 (5′-CCGCT- CGAGC GGTGGTCAGTCCTGCTCCTCGG-3′, *Xho* I recognition sites underlined), La (5′-ACTACGCATGCCTAAGTGC-3′) and 4 Lb (5′-CGCCCATACAGAAAAGA- AG-3′). These amplified products were sent to Beijing Genomics Institute (Shenzhen) for sequencing analyses.

### Mitochondrial Transformation Procedures

A biolistic PDS-1000 He particle delivery system (Bio-Rad, USA) was used for mitochondrial transformation. Gold particles (30 mg, 0.6 µM) were precipitated in a 1.5 ml Eppendorf tube. The pellet was resuspended in 1 ml of 70% ethanol and 1 ml of sterile water. The water was removed after gold particles were precipitated by centrifugation. Particles were resuspended with 5 µl of DNA (1 µg/µl). Subsequently, 50 µl of freshly prepared 2.5 M CaCl_2_ and 20 µl of 0.1 M spermidine was added to the DNA-gold particle mixtures. It was vortex mixed and centrifuged. The pellet was washed with 70% ethanol and the particles were suspended in 48 µl of 100% ethanol. *C. reinhardtii* strain *dum-1 mt^−^* CC-2654 was grown in liquid TAP medium up to the exponential phase (5∼6×10^6^ cells/ml). Cells were then collected and spread on TAP agar plates at 22°C in the light for 2 days. 8 µl of DNA particle suspension was loaded into the biolistic apparatus, and each plate was bombarded at 1,350 psi of helium. After bombardment, the cells were maintained on 1% TAP agar plate containing 100 µg/ml ampicillin and 3 µg/ml Zeocin in the light at 22°C for one day and then in darkness at 22°C for 7–8 weeks [1], [5], [6].

### Southern Blot and RT-PCR Analysis

Genomic DNA was isolated by using the DNeasy kit (Takara, Japan). The purified DNA was digested with restriction enzymes and the digested DNA fragments were separated on a 0.8% agarose gel. The DNA in the gel was blotted onto Hybond N+ (Amersham Biosciences) and probed with a random-primed *ble* probe using DIG high prime DNA labeling and detection starter kit I (Roche) according to the manufacturer's instructions.

Total RNA was isolated using SV Total RNA Isolation System (Promega, USA). A total of 2 µg of RNA was used to synthesize the first strand cDNA using Protoscript M-MuLV Taq RT-PCR Kit (NEB, USA), as per the manufacturer's instructions. The mRNA levels of *Sh ble*, *cob*, *nd4*, *nd5*, *cox1* and *nd2* were determined with RT-PCR using specific primers. The *Sh ble* primers were B1/B2. The o*ob* primers were 5′-ACCGTGGGCAAACTGAGTCTCCA-3′ (forward) and 5′-CCCAGCTAACCC ATATAGCACCCCA-3′ (reverse). The *nd4* primers were 5′- GCAGGACCACGT GGCAAACCA-3′ (forward) and 5′-CCCTTGCCATTTTCAGCCAAAGCG-3′ (reverse). The *nd5* primers were 5′- TCGAAACCCCAGCGAGAGCCA-3′ (forward) and 5′-TTGGCCGCTATTTGCGCTGA-3′ (reverse). The *cox1* primers were 5′- TGCCAGCCCTATTCGGTGGT-3′ (forward) and 5′-TGGGGCACGCAAACC AGCTAC-3′ (reverse). The *nd2* primers were 5′- TGCGCAACGGCCATGTG TCTT-3′ (forward) and 5′-CGGTTCGCTGATACATGGCGCTAA-3′ (reverse). The expression of *rrnL7* was used for internal control. The *rrnL7* primers were 5′-TAC CGTACTGCAAACCGACT-3′ (forward) and 5′-GATAAAATGTTACCCTGG-3′ (reverse). The PCR conditions for all amplifications were: 50 µL final volume, initial denaturation: 2 min at 95°C; 30 cycles of 30 s at 94°C, 30 s at 58°C, 30 s at 72°C. A final 8 min extension cycle performed at 72°C was used for PCR amplification [13]

### Northern Blot Analysis

Total RNA of MT-B and CC-2654 was fractionated in a 1.2% formaldehyde-agarose gel and transferred to Hybond N+ membrane. Northern blot hybridization was performed with the *Sh ble* probe as per the manufacturer's instructions.

### Western blot analysis

For detection of the BLE protein [13], total soluble protein was isolated from 20 ml cultures of *C. reinhardtii* grown to 5×10^6^ cells/ml. Cells were then collected by centrifugation at 5,000 g for 10 minutes and lysed by sonication in buffer containing 20 mM Tris-HCl, 10 mM EDTA, 50 mM NaCl, pH 7.4, and 1.0 mM phenylmethylsulfonyl fluoride. The cell extract was centrifuged at 19,000 g for 30 minutes. Protein samples were boiled for 5 minutes, separated by 10% SDS–PAGE gel and transferred electrophoretically onto polyvinylidene difluoride membranes (Millipore, Bedford, MA). The blots were blocked with 5% bovine serum albumin and treated with rabbit anti-*sh ble* antibodies (Cayla,France) at a dilution of 1∶5000. After washing, the blot was incubated with alkaline phosphatase conjugated goat anti- rabbit IgG secondary antibody (Proteintech Group, USA) and developed with BCIP/NBT alkaline phosphatase substrate solution (Promega, USA).
